# The Natural *Ficus carica* L. (fig) Extract as an Effective Prophylactic Antibacterial Agent for Inflammation-Related Infections

**DOI:** 10.3390/life13122356

**Published:** 2023-12-16

**Authors:** Junyoung Kim, Donghwan Lee

**Affiliations:** 1 Department of Bio-Nano System Engineering, College of Engineering, Jeonbuk National University, Jeonju 53896, Republic of Korea; kjy7148@naver.com; 2 Division of Mechanical Design Engineering, College of Engineering, Jeonbuk National University, Jeonju 53896, Republic of Korea; 3 Hemorheology Research Institute, Jeonbuk National University, Jeonju 54896, Republic of Korea

**Keywords:** mass spectrometry, deacetylation, eugenol, acetyleugenol, *Klebsiella pneumoniae*

## Abstract

*Klebsiella pneumoniae* (*K. pneumoniae*) is a multidrug-resistance Gram-negative organism responsible for carbapenem-resistant infections. These challenges have inspired studies on the use of natural products as alternatives to conventional drugs. The aim of this study was to analyze the antibacterial and antioxidant effects of *Ficus carica* L. (fig) branch extracts and to perform in vivo animal experiments to better understand the absorption mechanisms of the antibacterial components during the digestion process after oral administration. The antibacterial components of the fig branch extracts were analyzed via gas chromatography-mass spectrometry (GC-MS). An in vivo animal study and liquid chromatography-triple quadrupole-tandem mass spectrometry (LC-QQQ-MS/MS) analyses were performed to analyze the deacetylation reactions of the fig extracts after oral administration in mice. Ultimately, the antibacterial effects of the fig extracts increased with the fractional distillation time. The fig extracts showed excellent antibacterial effects against *K. pneumoniae*, as well as *Escherichia coli* (*E. coli*), *Staphylococcus aureus* (*S. aureus*), and *Pseudomonas aeruginosa* (*P. aeruginosa*). The three antibacterial and antioxidant components of the fig extracts were revealed to be eugenol, acetyleugenol, and psoralen. Interestingly, in this study, we identified acetyleugenol in the phenolic compounds of the fig extract for the first time. Through in vivo animal testing, we observed the deacetylation reaction of acetyleugenol to eugenol in the fig extract as digestion proceeded in the internal organs of the mice after oral administration. The results of this study suggest the use of natural fig extract as an effective therapeutic and prophylactic antibacterial agent for inflammation-related infections with a wide variety of biomedical applications.

## 1. Introduction

*Klebsiella pneumoniae* (*K. pneumoniae*) strains are highly virulent and associated with toxic infections including pneumonia, cartilage tissue infection, urinary tract infections, and bacteremia [1,2]. *K. pneumoniae* is a multidrug-resistance Gram-negative organism [3] that causes challenges related to beta-lactam antibiotic resistance [4]. *K. pneumoniae* produces extended-spectrum β-lactamase (ESBL), an enzyme widespread among Gram-negative bacteria that hydrolyzes the beta-lactam rings of antibiotics [4].

*K. pneumoniae* is generally considered to contain one of the resistant genes for antibiotics that are multidrug-resistant [3]. This factor has rendered third-generation cephalosporins ineffective and has made carbapenems an option for *K. pneumonia*. However, recent studies have shown that *K. pneumoniae* is responsible for carbapenem-resistant infections, consequently limiting the use of carbapenems for beta-lactam-resistant *K. pneumonia* infections [2,5]. This resistance has inspired the development of natural products with significant prophylactic efficacy, specially made to adjust the composition of beneficial bacteria in the gut [6,7]. Currently, there is a growing preference for natural products over conventional pharmaceutical products due to their naturally pure and active chemical ingredients with high stability and abundance [8].

A wide range of natural products are physiologically active, with antibacterial, anti-inflammatory, and antioxidant properties. These products are often used in medicine, nutrition, pharmaceuticals, and alternative medicine [9]. Commendably, several natural compounds have been reported to prevent antibiotic resistance, making them important therapeutic candidates for bacterial infections [10]. However, while medicinal plants have shown high pharmacological activity, there is also growing concern about the toxicity and side effects of hundreds of dietary herbs and vegetables, especially for patients attempting to treat different unproven diseases simultaneously [11].

*Ficus carica* L. (fig) is mainly composed of phenolic compounds, vitamins, minerals, sugar, proteins, amino acids, and organic acids; this fig is used for medicinal and food purposes [12,13,14,15,16]. In previous studies, the trunks, branches, and leaves of the fig tree were reported to have a variety of biological properties, including anticancer, antibacterial, antioxidant, and anti-inflammatory properties [17]. Two varieties of dried figs exhibited potential as natural sources of antioxidant and antimicrobial compounds, indicating significant correlations between phytochemical content and both antioxidant and antimicrobial activities [18]. Fig extract (an edible fruit) was utilized as both a capping and reducing agent to synthesize calcium oxide nanoparticles (CaONPs), suggesting the potential therapeutic value of these nanoparticles as antibacterial and antibiofilm agents in medications [19]. Interestingly, the synergistic antimicrobial activity of plant extracts and cefixime was assessed in multi-drug-resistant clinical isolates, particularly highlighting the significant synergism of ethyl acetate and methanol extracts with cefixime against both Gram-positive and Gram-negative resistant strains [20]. Fig tree extract was also used as an anti-inflammatory antispasmodic for cardiovascular, respiratory, and metabolic disorders [21]. Moreover, an aqueous extract of fig leaves was shown to have a blood-sugar-reducing effect in an animal study [22].

Although there are many studies reporting the medicinal value of fig tree extracts, the mechanism of their medicinal effects remains understudied [16]. Therefore, using in vivo animal experiments, the purpose of this study was to precisely analyze the antibacterial components contained in fig branch extracts, experimentally verify their antibacterial effects against *K. pneumoniae*, and investigate their absorption processes during digestion when orally administered.

## 2. Materials and Methods

### 2.1. Preparation of the Fig Extracts from Branches

Mature fig tree branches (1 kg) were cut into small pieces, pulverized into a fine powder, and successively extracted using chloroform (CHCl_3_, fraction I) and methanol (MeOH, fraction II). During extraction, the liquid’s temperature was maintained at 17 ± 2 °C for 48 h. Then, all the extracts were filtered. Next, the solvents were evaporated to dryness under reduced pressure. The extract, concentrated with CHCl_3_ and MeOH, was filtered to remove fig residue. The filtrate was heated, and then the vaporized gas was cooled through a liquefaction process. Here, the product discharged through the tube was fractionally distilled at 5 min intervals up to 35 min. Finally, the distilled fig extracts were stored at a temperature between 4 and 6 °C. Fig branches were collected in September 2019 from the Samho farm of Yeongam-gun in South Korea. The identity of the figures was confirmed by an expert. However, due to limitations in sample documentation, the voucher specimen number for the fig branch sample used in this study is not available. Future efforts will be made to address and provide these details.

### 2.2. Gas Chromatography-Mass Spectrometry (GC-MS) Analysis

Gas chromatography-mass spectrometry (GC-MS) analysis was performed on the fig extracts using a gas chromatograph combined with a mass spectrometer (QP2010, Shimadzu, Kyoto, Japan) at an ionization voltage of 70 eV. The analysis was conducted using a Restek column (0.25 mm, 30 m; XTI-5) in temperature programming mode. The initial temperature of the column was set to 70 °C for 3 min and increased linearly at a rate of 10 °C/min to 300 °C. Then, the temperature was held at 300 °C for 5 min. The temperature of the GC-MS interface was 290 °C, and the injection port was set to 280 °C. A scan mode of 45 to 500 *m*/*z* (mass-to-charge ratio) was used to capture a comprehensive spectrum of compounds in the fig extract sample, allowing the detection and identification of a wide variety of molecules with different masses [23,24]. The helium carrier gas flow rate was 1.0 mL/min. The presence of major components in the fig branch extracts was verified by comparing standards and spiking.

### 2.3. Antibacterial Test

A liquid Luria–Bertani (LB) medium containing 1.0 g beef extract, 0.5 g NaCl, 0.5 g peptone, and 0.1 L distilled water (DW) was prepared and autoclaved at 15 psi and 125 °C for 15 min. The reference strains of *Staphylococcus epidermidis* (*S. epidermidis*) (KCTC 13170) were obtained from the Korean Collection for Type Cultures (KCTC, Daejeon, Republic of Korea). The bacteria were cultured in a liquid LB medium for 24 h at 30 °C in a shaking incubator at 200 rpm. After culturing bacteria in the liquid LB medium, the culture was evenly spread on a solid LB agar plate.

Using fig extract concentrate samples prepared at different fractional distillation times (10, 15, 20, 25, 30, and 35 min), antibacterial tests were performed on *S. epidermidis*, a well-known microorganism that lives on the skin. Sterile filter paper disks (5 mm in diameter) were prepared, and 10 µL of fig branch extracts were evenly dispensed onto the paper disks. Then, the disks were placed on the surface of an agar plate using sterile forceps. After incubating for 48 h at 30 °C, the size of the clear zone where the bacteria did not reproduce around the paper disks was evaluated. Ampicillin was used as a standard in the antibacterial test. Among the fig extract samples at different fractional distillation times, the sample with the strongest antibacterial effect was chosen, and additional antibacterial experiments were conducted on *K. pneumoniae*, *Escherichia coli* (*E. coli*), *Staphylococcus aureus* (*S. aureus*), and *Pseudomonas aeruginosa* (*P. aeruginosa*), respectively. Strains known to have multi-drug resistance were selected for this study [25,26].

### 2.4. Antioxidant Capacity Assays

A hydrogen peroxide scavenging assay was prepared by diluting fig extracts to concentrations ranging from 10^−4^ to 10^−6^ (mg/mL). Initially, 300 µL of fig branch extract was combined with 600 µL of a 0.1 M phosphate buffer (pH 7.4), supplemented with 45 mM of hydrogen peroxide. The mixture was allowed to react for 10 min, and the absorbance (ABS) was measured at 230 nm using a UV-Vis spectrophotometer (Libra S70, BioChrom, Cambridge, UK). The scavenging activity of hydrogen peroxide (%) was determined and calculated using the following equation [27,28]:

Hydrogen peroxide scavenging activity (%) = [(ABS_Control_ − ABS_Sample_)/ABS_Control_] × 100. (1)

To evaluate the 2,2-diphenyl-1-picrylhydrazyl (DPPH) radical scavenging activity, 24 mg of DPPH powder was dissolved in 100 mL of methanol and stored at −20 °C. The mixed DPPH solution was adjusted to an absorbance of 0.98 ± 0.02 at 517 nm prior to use. Next, 1.5 mL of DPPH solution was diluted to concentrations of 10^−2^, 10^−3^, and 10^−4^ (mg/mL) of the fig branch extracts, and the reaction occurred in the dark for 30 min. The absorbance (ABS) was then measured using a spectrophotometer (Libra S70, Biochrom, UK) at 517 nm. The DPPH radical scavenging activity of each fig extract sample was calculated using the following equation [27,28]:

DPPH radical scavenging activity (%) = [(ABS_Control_ − ABS_Sample_)/ABS_Control_] × 100. (2)

A calibration curve was prepared using the standard Trolox substance under the same conditions. The value of the sample was substituted and represented as the TEAC (Trolox equivalent antioxidant capacity) μmol/mL (sample). Ascorbic acid was used as a standard in the hydrogen peroxide scavenging activity study, and Trolox was used as a standard in the DPPH study.

### 2.5. In Vivo Animal Study

To evaluate changes to the chemical components of the fig branch extracts during digestion, an animal study was conducted using six-week-old Balb/c mice (n = 30, female). The mice were obtained (Han-il Laboratory Animal Center, Wanju, Republic of Korea) and housed under specific-pathogen-free (SPF) standard environmental conditions (22 ± 2 °C temperature, 45 ± 5% humidity, and 12 h of light/12 h of dark cycle with readily available water and food) in an animal room. The Institutional Animal Care and Use Committee (IACUC) at the Jeonbuk National University Laboratory Animal Research Center approved all experimental procedures and animal handling. Efforts were made to minimize any pain, distress, or discomfort experienced by the animals, which included the use of anesthesia, analgesia, or other appropriate methods to alleviate suffering during procedures.

The mice were randomly divided into two groups based on sampling time intervals after oral administration at 1 h and 2 h after delivery of the oral injection. These specific time points were determined based on the initial experiment, considering the deacetylation time of acetyleugenol after oral injection. Additional analysis was performed after 2 h of oral injection, taking into account the time needed for the deacetylation and conversion of acetyleugenol to eugenol.

The fig branch extracts (4 mg plant extracts + 0.2 mL water with 8% glucose) were administered orally to the mice using a microinjector. A bronchoalveolar lavage fluid (BALF) sample was collected by inserting a catheter into the lungs of the mice and injecting 10–20 mL of sterile physiological saline [29]. Blood samples were obtained from the cardiac vessels entering the right atrium of the mice. Lung tissues were sliced and collected. All samples were stored for 24 h at −40 °C.

### 2.6. Quantitative Analysis of Eugenol and Acetyleugenol Using LC-QQQ-MS/MS

For the quantitative analysis of eugenol and acetyleugenol, liquid chromatography-triple quadrupole-tandem mass spectrometry (LC-QQQ-MS/MS) with an electrospray ionization (ESI) source (Agilent 6410B, Agilent Technologies, Wilmington, NC, USA) was conducted on the blood, lung tissue, and BALF samples of the mice. BALF samples were used as a stock solution without dilution; meanwhile, for the blood samples, 100 µL of blood was diluted with 900 µL of MeOH. Lung samples were crushed in distilled water and then diluted with 900 µL of MeOH. To further subdivide the tissue sample, all lung tissue samples were subjected to a sonication process. The supernatant was obtained from the separated sample after centrifugation and placed into a 1 mL vial for quantitative analysis.

Then, 5 µL aliquots of the treated samples were injected into a high-performance liquid chromatography (HPLC) system (1200 Series LC, Agilent Technologies, Wilmington, NC, USA). The system was equipped with a Phenomenex Synergi Hydro-RP column (4 µm, 80 Å, 150 × 2 mm) and maintained at 30 °C. ESI was run at +3000 V. The temperature at the source was 380 ˚C. The source offset, capillary voltage, and cone voltage were set to 30 V, 3 kV, and 30 kV, respectively. Solvent evaporation cone gas flow and gas flow were set to 150 L/Hr and 650 L/Hr, respectively, at a nebulizer pressure of 15 bars. The samples were separated using Buffer A (formic acid of 0.1% in distilled water) and Buffer B (formic acid of 0.1% in acetonitrile) and pumped through the chamber of the ESI (0.5 mL/min) for 20 min. Both the fragmentor voltage and collision voltage were set to 15 V. The multiple-reaction monitoring mode was used to track the ions by tracking the transition pairs of eugenol (165.08 → 136.85 *m*/*z*) and acetyleugenol (207.04 → 188.95 *m*/*z*). The data were obtained using the MassHunter Software, Version B.04.00 (Agilent Technologies, Wilmington, NC, USA).

### 2.7. Statistical Analysis

Statistical analysis of our data was performed using Microsoft Excel 2021. To compare the antibacterial effects according to different fractional times, a one-way analysis of variance (ANOVA) was performed on the measured zone of the inhibition values. A *p*-value less than 0.05 was considered statistically significant. Where applicable, results were presented as the means and standard deviations.

## 3. Results

**Figure 1:**
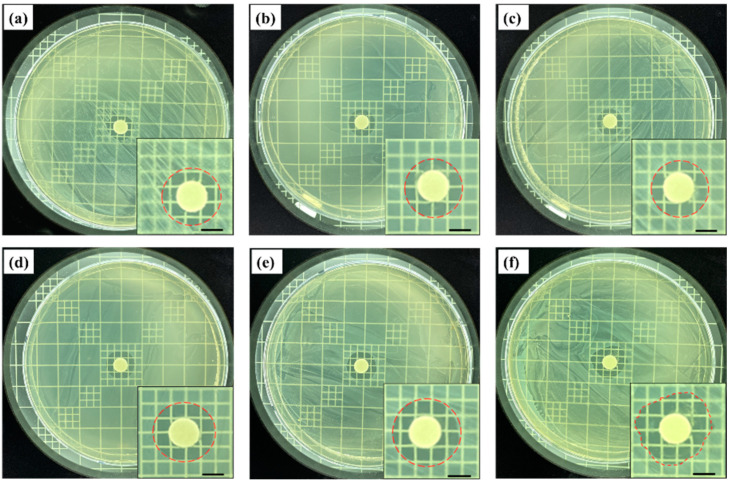

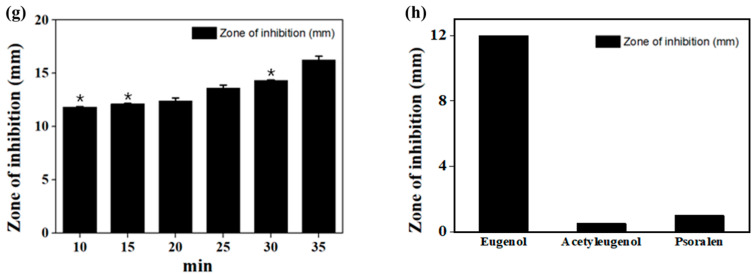
Antibacterial effects of the fig branch extract against *Staphylococcus epidermidis* (*S. epidermidis*) at different fractional distillation times of (**a**) 10 min; (**b**) 15 min; (**c**) 20 min; (**d**) 25 min; (**e**) 30 min; and (**f**) 35 min. Scale bars (**a**–**f**): 5 mm. The measured zone of inhibition after the antibacterial test using (**g**) the fig branch extracts at different fractional distillation times (* *p* < 0.05) and (**h**) pure eugenol, acetyleugenol, and psoralen for comparison with fig extracts.

Figure 1 shows the results of antibacterial tests against *Staphylococcus epidermidis* (*S. epidermidis*) using the fig branch extracts obtained at different fractional distillation times. After 48 h of incubation, the antibacterial effects of the fig branch extracts distilled for 10 min covered an antibacterial range of 11.9 mm, as shown in Figure 1a. As the fractional distillation time was further sustained for 15, 20, 25, 30, and 35 min, the antibacterial ranges were widened to 12.2 mm, 12.4 mm, 13.6mm, 14.4 mm, and 16.3 mm, respectively, as shown in Figure 1b–f. This result indicated that the antibacterial effects of the fig branch extracts increased with a longer fractional distillation time. As a result, it was confirmed that the later the fig extract was released, the greater the antibacterial activity became.

We also performed control experiments against *S. epidermidis* using pure eugenol, psoralen, and acetyleugenol to compare their antibacterial properties with those of the fig extracts. As shown in Figure 1h, when used alone, pure eugenol showed the strongest antibacterial effects, followed by psoralen and acetyleugenol. Comparing Figure 1g,h confirms that the fig extract offered stronger antibacterial effects than pure compounds. This likely occurred because, in the case of the fig extract, the antibacterial effects of the three compounds were combined and worked simultaneously.

As shown in Table 1, with the fig branch extracts obtained at a fractional distillation time of 35 min, additional antibacterial tests were performed against *K. pneumoniae*, *E. coli*, *S. aureus*, and *P. aeruginosa* using paper disk methods. The fig branch extracts (35 min of fractional distillation time) demonstrated an effective inhibition area against *K. pneumoniae* (24.5 ± 1 mm), *E. coli* (15.5 ± 0.75 mm), *S. aureus* (17 ± 0.5 mm), and *P. aeruginosa* (8.25 ± 0.38 mm). Interestingly, these extracts offered the most effective antibacterial effects against *K. pneumoniae*, which is a bacterium that induces inflammation. These findings indicated that the fig branch extracts offered better antibacterial properties than the alternatives, especially against inflammatory bacteria.

**Figure 2:**
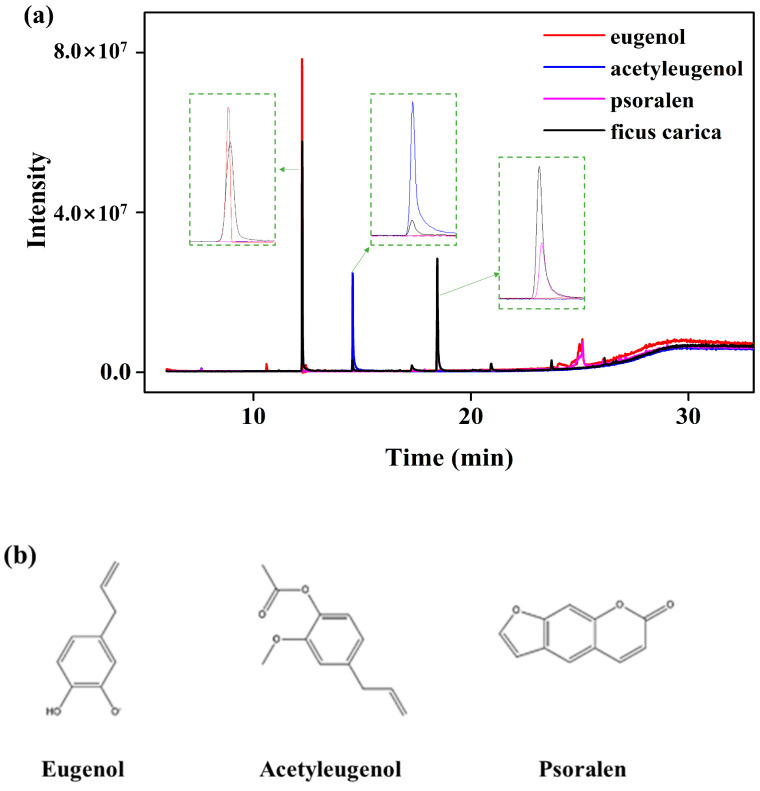
(**a**) Gas chromatography-mass spectrometry (GC-MS) of the fig branch extracts (black line) and standard samples of eugenol (red line), acetyleugenol (blue line), and psoralen (pink line). (**b**) Chemical structures of the isolated eugenol, acetyleugenol, and psoralen compounds.

The results of the GC-MS analyses of the fig branch extracts are shown in Figure 2. Initially, a total of seven compounds were detected. Among them, five compounds were included in the list, starting with those featuring the highest concentration, including the newly discovered acetyleugenol. Interestingly, among the five peaks, the three peaks with eugenol, psoralen, and acetyleugenol are known to have excellent antibacterial and antioxidant activities. The value at each peak was matched with the corresponding library spectrum. The peaks were double-confirmed via GC-MS using standard material.

Table 2 shows the high-resolution mass spectrometry characterization and identification of data for the five major peaks in the MS chromatograms of the fig branch extracts. Among the antibacterial- and antioxidant-related three peaks, the amount of eugenol was the highest (peak area 50.32%), followed by psoralen (peak area 38.97%), whereas the amount of acetyleugenol was relatively small (peak area 2.89%). The GC/MS spectra of the available standard compounds were confirmed by mass spectrometric identification of individual compounds. When standards were not available, tentative identification was made using published literature and the PubChem database.

**Figure 3:**
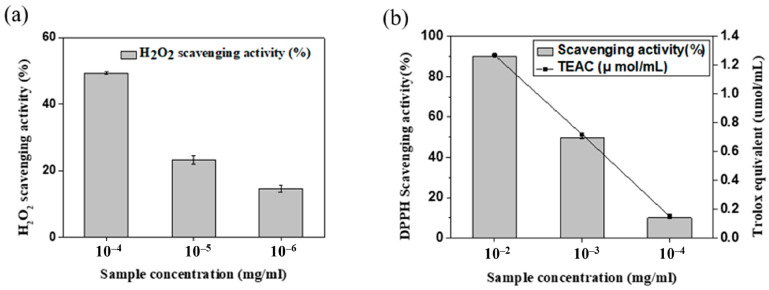
(**a**) H_2_O_2_ scavenging activity and (**b**) DPPH free radical scavenging activity of the fig branch extracts.

The antioxidant activities of phenolic compounds in the fig branch extracts were confirmed using two different biochemical analysis methods: a hydrogen peroxide scavenging assay and a DPPH free radical scavenging activity assay. As shown in Figure 3a, when the fig branch extract was diluted to concentrations of 10^−4^, 10^−5^, and 10^−6^ (mg/mL), the H_2_O_2_ scavenging activities were observed to be 49.35 ± 0.29%, 26.21 ± 1.25%, and 14.55 ± 1.00%, respectively. As shown in Figure 3b, the scavenging activity against DPPH free radicals was investigated using fig branch extracts diluted to concentrations of 10^−2^, 10^−3^, and 10^−4^ (mg/mL). At a concentration of 10^−2^ (mg/mL), the DPPH free radical scavenging activity of the fig branch extracts was 90.07 ± 0.15%. Meanwhile, when diluted to concentrations of 10^−3^ and 10^−4^ (mg/mL), the antioxidant activities decreased to 49.89 ± 0.65% and 9.99 ± 0.38%, respectively.

**Figure 4:**
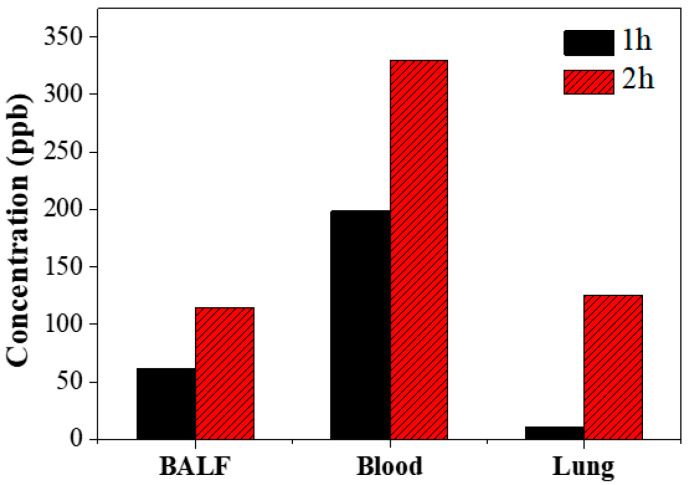
The concentration of eugenol in each mouse organ at 1 and 2 h following oral injection of fig branch extract mixtures.

Figure 4 shows the concentrations of eugenol in BALF, blood, and lung tissue samples of mice, which were measured 1 h (black) and 2 h (red) after oral injection of the fig branch extract mixtures. One hour after oral injection, the concentrations of eugenol were 61.6 ppb, 198.5 ppb, and 26.5 ppb in BALF, blood, and lung tissue samples, respectively. Two hours after oral injection, the concentrations of BALF, blood, and lung tissue samples increased to 114.7 ppb, 330.2 ppb, and 124.9 ppb, respectively. Notably, acetyleugenol was not detected in any of the samples one or two hours after oral injection. These findings theoretically suggest that acetyleugenol could be consistently deacetylated even one hour after oral administration as digestion occurs in the internal organs.

## 4. Discussion

Through the GC-MS analysis, we confirmed that the three antibacterial components in the fig branch extracts were phenolic compounds such as eugenol, acetyleugenol, and psoralen. Interestingly, in this study, we identified acetyleugenol among the phenolic compounds of the fig extract for the first time. Although the content of acetyleugenol (peak area 2.89%) was much lower than that of eugenol (peak area 50.32%), this result was considered a remarkable discovery.

When comparing the antibacterial efficacy of eugenol, psoralen, and acetyleugenol, eugenol was found to have the strongest antibacterial effect, followed by psoralen. However, concerns have been raised regarding the toxicity of eugenol and psoralen. On the other hand, acetyleugenol has the advantage of exhibiting antibacterial effects while maintaining low toxicity [30,31,32,33,34].

Eugenol is an alkylphenol known to be metabolized into quinone methide intermediates by cytochrome P450 and peroxidase enzymes. Several studies on hepatocytes and other cell types have linked this quinone methide to the toxicity mechanism of eugenol [35,36]. Acetyleugenol has a common acetyl group (C_2_H_3_O), which was shown to be responsible for antibacterial activity [31]. It was found that by acetylating acetyl derivatives from eugenol and thymol compounds, it is possible to synthesize acetyleugenol and acetylthymol, which are novel structures of active compounds with low toxicity but high antibacterial activity [37].

In this study, the amount of eugenol was the highest, followed by psoralen, while the amount of acetyleugenol was relatively small. We sought to compare the amount of phenolic compounds (i.e., eugenol and acetyleugenol) extracted in our study with the amount extracted from other plants in the literature to contextualize our findings and understand the relative abundance of these compounds in different plant sources. For example, eugenol and acetyleugenol were extracted from cloves, and eugenol was identified by comparing the retention time and peak appearance in the clove extract [38,39]. Psoralen was reported to be the most abundant in fig leaves, accounting for approximately 34% of all identified compounds [40]. However, the extraction concentrations were not explicitly specified in the literature, making it difficult to compare directly.

The antibacterial effects of the fig branch extracts were verified against diverse microorganisms, with strength in the following order: *K. pneumoniae*, *S. aureus*, *E. coli*, and *P. aeruginosa*. Notably, the extracts derived from fig branches offered the most effective antibacterial effects against the inflammatory bacteria *K. pneumoniae*. Also, the antibacterial effect of the fig branch extracts increased with a longer fractional distillation time. The antibacterial effect may have improved because the longer the extraction time, the higher the content of antibacterial phenolic compounds in the fig branch extracts. However, when the fractional distillation time exceeded 35 min, the antibacterial effect did not increase any further because the concentration of extractable components was limited.

The observed antibacterial effects, particularly against *K. pneumoniae*, might hold significant clinical implications due to the increasing prevalence of antibiotic resistance in this pathogen. Given the rising concern about treating infections caused by multidrug-resistant strains, the effectiveness of the antibacterial activity against *K. pneumoniae* is noteworthy. The observed antibacterial effects of the extracted compounds from fig branches could offer a promising avenue for the development of novel antibacterial agents.

The fig branch extracts were obtained using the solvent extraction method rather than the distillation method. In the extraction method, fig branch extracts obtained through distillation showed no antibacterial activity against *S. epidermidis*. On the other hand, fig branch extracts obtained via solvent extraction using chloroform and methanol showed antibacterial activity against *S. epidermidis*.

In addition, when applying the solvent extraction method, an initial attempt was made to fractionate the extract using distilled water (DW). However, in the case of fig branch extracts using DW, no antibacterial activity against *S. epidermidis* was observed. On the other hand, solvent-extracted fig extracts using chloroform and methanol showed excellent antibacterial activities against *S. epidermidis*. The absence of antibacterial substances in the distillate can be attributed to the nonpolar nature of these extracts, while the solvent employed is relatively polar and contains a significant amount of water. Fractionation further revealed that the active compounds featured relatively hydrophobic characteristics, as they eluted at a later stage in the gradient. These compounds may require extraction using highly nonpolar solvents [41].

Antioxidants play a crucial role in neutralizing free radicals, which are reactive molecules linked to various chronic diseases and aging. The presence of antioxidants in fig extracts suggests potential protective effects against oxidative stress-related conditions. The identified antioxidant compound in fig extracts offers a promising avenue for promoting health and preventing diseases by combating oxidative stress and inflammation.

Free radicals, in the form of hydroxyl radicals, peroxides, and superoxides, are known to damage tissues, contributing to aging and cancer cell growth through cell mutation [42]. To comprehensively assess the antioxidant properties of natural fig extracts, both hydrogen peroxide and DPPH scavenging assays were employed [43]. In this analysis, the phenolic compounds in fig extracts exhibited excellent antioxidant activities, suggesting the potential use of fig extracts as antioxidant candidates through their ability to suppress oxidant reactions and eliminate free radicals from the body.

In the in vivo animal experiment, oral injection of fig branch extracts, including eugenol and acetyleugenol, was conducted to investigate the digestion process of acetyleugenol as an antibiotic. Quantitative analysis using LC-QQQ-MS/MS revealed that there was no acetyleugenol in BALF, blood, or lung tissue samples one hour after oral administration. On the other hand, the concentration of eugenol gradually increased as digestion proceeded. These findings indicate that orally administered acetyleugenol with low toxicity might be converted to eugenol by following the deacetylation reaction occurring in the body.

To date, no studies have investigated the deacetylation of acetyleugenol to eugenol in the body. Nevertheless, it is anticipated that acetyleugenol undergoes deacetylation to form eugenol, given the observed increase in eugenol levels in the body. Analogous instances include processes involving histone deacetylases (HDACs) and chitin de-N-acetylases (CDAs) [44,45].

The absence of acetyleugenol after one hour was considered surprising based on prior knowledge, as this result suggested a rapid deacetylation process. Prior research indicated that the conversion of eugenol to acetyl eugenol is typically completed within 16 h, albeit in the opposite direction [46]. The absorbed acetyleugenol could be attributed to its continuous deacetylation during digestion in the internal organs, as evidenced by the sustained increase in eugenol levels over the two-hour period.

Additional studies are required to clarify the specific control mechanisms governing the deacetylation of acetyleugenol in the intestines and its subsequent impact on the release of eugenol in the body. Furthermore, histologic examinations and hematologic analyses of the organs are necessary to assess the levels of eugenol and acetyleugenol retained in each organ. These fundamental studies will contribute valuable insights, potentially offering guidelines for the optimal oral dosage of phenolic compounds extracted from fig branches as a potential antibacterial agent.

## 5. Conclusions

This study demonstrated the chemical diversity and antibacterial efficacy of fig extracts and proposed oral administration as a possible therapeutic candidate for bacterial infections. GC-MS analysis showed that the fig extracts mainly consisted of three antibacterial compounds: eugenol, acetyleugenol, and psoralen. The amount of eugenol was the highest, followed by psoralen and acetyleugenol. In particular, acetyleugenol was confirmed to be included among the phenolic compounds of the fig extracts for the first time. The excellent inhibition of *K. pneumoniae* demonstrated that fig extracts represent an effective antibacterial option for drug-resistant bacteria. Using both hydrogen peroxide scavenging activity assays and DPPH free radical scavenging activity assays, the antioxidant effects of the fig extracts were successfully evaluated. Through an in vivo study and LC-QQQ-MS/MS analysis, the deacetylation reaction of acetyleugenol to eugenol was successfully detected. Acetyleugenol might be deacetylated into eugenol through metabolism, subsequently enhancing antibacterial activity against inflammation-causing bacteria. Therefore, the oral administration of fig extracts might be a potential therapeutic and prophylactic antimicrobial option for *K. pneumoniae* strains, as well as for inflammation-causing infections.

## Figures and Tables

**Table 1:** Antibacterial activities of the fig branch extracts obtained at a fractional distillation time of 35 min against various microorganisms.

No.	Microorganism	Zone of Inhibition (mm)
1	*Klebsiella pneumoniae*	24.5 ± 1
2	*Escherichia coli*	15.5 ± 0.75
3	*Staphylococcus aureus*	17 ± 0.5
4	*Pseudomonas aeruginosa*	8.25 ± 0.38

**Table 2:** Results of GC-MS analysis for the fig branch extracts.

No.	Peak Name	R/T (min)	Molecular Formula	MW	Peak Area%	Identification (Literature)
1	Eugenol	12.25	C_10_H_12_O_2_	164	50.32	^a^
2	Acetyleugenol	14.56	C_12_H_14_O_3_	206	2.89	^a^
3	Psoralen	18.45	C_11_H_6_O_3_	186	38.97	^a^
4	Methoxsalen	20.93	C_12_H_8_O_4_	216	3.01	^a^
5	Matridin-15-one	23.70	C_15_H_24_N_2_O	248	2.82	https://pubchem.ncbi.nlm.nih.gov/compound/91466 (accessed on 5 January 2023)

^a^ identified by comparing it with standard compounds.

## Data Availability

All data used to support the findings of this study are available from the corresponding author upon request.
